# Plasma cholinesterase activity in children with infective endocarditis

**DOI:** 10.1186/1749-8090-8-S1-P177

**Published:** 2013-09-11

**Authors:** LJ Popovic, S Alavuk, T Kovacevic

**Affiliations:** 1 Department of Anesthesiology, Reanimatology and Intensive Care, Children's Hospital Zagreb, Zagreb, Croatia

## Background

Although pre-existing heart disease in children is the most frequent predisposing factor for infective endocarditis (IE), we reporte 6 children of IE with no apparent pre-existing cardiac disease.

## Methods

Our study included six patients with acquired infection from central catheter; the microbial pathogens were Streptococcus viridans (n=3) and Staphyloccocus aureus (n=3). The diagnosis of IE was based on positive blood culture (2 positive cultures of blood samples drawn > 12 hours), evidence of endocardial involvement (positive ECHO), measuring raised erythrocyte sedimentation rate and plasma cholinesterase (PChE) activity. We measured PChE activity (µmol min^-1^ml^-1^) in children with IE (group A, n=6) and in IE-free children (group B, n=10). For determination of PChE activity vein blood samples were collected and stored at -20°C until analyzed. PChE activity was determined by the spectrophotometric method of Ellman using butyryltiocholine as the substract (Sigma Chemical Co., St.Luis, Mo, USA). Statistical analysis was made by Student’s t-test.

## Results

In group A of examined children with IE we measured decreased PChE activity (1.89 ± 0,24) relation to patients of control group B (3,97 ± 0,20).

## Conclusion

Our study indicates that PChE activity can be also an important parameter for the diagnosis of IE.

